# Lactoferrin bridges antimicrobial and healing responses in *Staphylococcus aureus* skin infections

**DOI:** 10.3389/fmicb.2025.1753483

**Published:** 2026-01-23

**Authors:** Katarzyna Kosznik-Kwaśnicka, Urszula Leszczyńska, Lidia Piechowicz

**Affiliations:** 1 Department of Medical Microbiology, Faculty of Medicine, Medical University of Gdańsk, Gdańsk, Poland; 2 Faculty of Medicine, Medical University of Gdańsk, Gdańsk, Poland

**Keywords:** antimicrobial resistance, lactoferrin, MRSA, skin infections, *Staphylococcus aureus*, wound infections

## Abstract

Staphylococcus aureus is a leading cause of skin and wound infections worldwide, with methicillin-resistant strains (MRSA) posing a persistent clinical challenge due to antibiotic tolerance and biofilm formation. Lactoferrin, an iron-binding glycoprotein abundant in mammals’ secretions and neutrophil granules, has emerged as a promising multifunctional agent that could help manage staphylococcal skin and wound infections, as it combines direct antimicrobial activity with immunomodulatory and tissue-repair effects. This mini-review aims to synthesize current evidence on the role of lactoferrin in the prevention and treatment of staphylococcal skin and wound infections, focusing on its antimicrobial mechanisms, modulation of host responses, and therapeutic applications. *In vitro* studies demonstrate that lactoferrin inhibits *S. aureus* growth through iron sequestration and membrane disruption, and it can also disrupt biofilm formation and persistence. Additionally, experiments showed that lactoferrin modulates inflammation, reduces oxidative stress, and promotes fibroblast migration and collagen deposition, facilitating wound closure. Lactoferrin incorporated into hydrogels, films, or nanocarriers enhanced antibacterial activity and synergized with antibiotics or bacteriophages in preclinical models. Nonetheless, variability in dosing, formulation, and study design limits cross-study comparisons, and potential bacterial resistance mechanisms remain underexplored. Therefore, further controlled and standardized studies are needed in order to optimize clinical translation and integration into modern wound care.

## 1 Introduction

*Staphylococcus aureus* is a dominant cause of skin and soft tissue infections (SSTIs), ranging from superficial infections such as impetigo to severe conditions such as cellulitis or Staphylococcal Scalded Skin Syndrome (SSSS) (Del Giudice, 2020). The clinical threat posed by this microorganism has been drastically amplified by the escalating crisis of antimicrobial resistance, particularly with the widespread prevalence of Methicillin-Resistant *S. aureus* (MRSA). MRSA strains are responsible for both community-acquired and hospital-acquired skin infections, challenging existing therapeutic protocols (Bassetti et al., 2009; Almuhayawi et al., 2023). A key factor driving the chronicity of *S. aureus* infections is the pathogen’s ability to form biofilms: complex, structured bacterial communities encased in a self-produced polymeric matrix that creates an environment that shields the bacteria from host immune defenses and systemic antibiotics (Dauros-Singorenko et al., 2020; Peng et al., 2023). Biofilm formed at the site of infection may lead to persistent colonization and delayed healing. Furthermore, bacterial cells may detach from mature biofilm, establishing new infection sites or causing systemic reactions, such as sepsis (Minasyan, 2019). Therefore, effective management of infected skin, soft tissue, or wounds requires agents that can both eradicate the pathogen and stimulate the host’s restorative mechanisms.

Lactoferrin (Lf) is an iron-binding glycoprotein central to innate immunity, found in milk, tears, saliva, and neutrophil granules. Although its antibacterial and antiviral properties were the subject of research for years, the scientific interest in lactoferrin peaked during the COVID-19 pandemic, as it was shown that it can act as a protective agent against the SARS-CoV-2 virus as well as alleviate symptoms of an ongoing infection (Bolat et al., 2022; Palacios-Rosas et al., 2025). Lactoferrin’s antimicrobial activity arises primarily from high-affinity iron sequestration, depriving pathogens of this essential nutrient, and from direct interactions with microbial cell walls that result in permeability changes and cell lysis (Aguila et al., 2001; Kell et al., 2020). Additionally, Lf and its derived peptides can inhibit biofilm formation, neutralize bacterial toxins, and enhance host immune responses (Ammons and Copié, 2013; Drago-Serrano et al., 2017). These multifaceted properties make Lf a key component of innate immunity with broad activity against bacteria, fungi, and viruses, including antibiotic-resistant strains of *Staphylococcus aureus*.

Given the urgent need for novel strategies to combat antibiotic-resistant *S. aureus* and its biofilm-associated persistence in skin infections, lactoferrin emerges as a promising multifunctional therapeutic candidate that simultaneously inhibits bacterial growth, attenuates virulence, and promotes tissue repair. However, despite a growing body of experimental and clinical evidence, the therapeutic relevance, delivery approaches, and mechanistic nuances of lactoferrin in the context of *S. aureus*-mediated skin pathology remain incompletely defined. This mini-review synthesizes current experimental evidence on the antimicrobial, antibiofilm, immunomodulatory, and wound-healing roles of lactoferrin in *S. aureus* skin and wound infections.

## 2 Lactoferrin activity against *Staphylococcus aureus*

### 2.1 Molecular basis of lactoferrin activity against *S. aureus*

The three studies by Naidu et al. established the early foundational understanding of how *S. aureus* interacts with human and bovine lactoferrin. In their first clinical isolate study, the authors demonstrated that lactoferrin can bind to *S. aureus* strains, though the strength of this relation was strain-dependent. The binding was shown to be specific and saturable, suggesting the presence of dedicated bacterial surface components that recognize Lf (Naidu et al., 1991a). Building on this, the study published a year later identified and characterized a distinct human lactoferrin-binding protein on the surface of *S. aureus*. The study further showed that this protein varies in expression across strains, helping to explain the heterogeneity observed in earlier work (Naidu et al., 1992). The third study extended these findings to bovine mastitis isolates, comparing their binding of lactoferrin with the binding of subepithelial matrix proteins. The authors observed that bovine strains also displayed lactoferrin-binding activity, though the strength and pattern of binding differed from those of human clinical isolates. This variability suggested host-associated adaptations in how *S. aureus* engages with lactoferrin across different environments (Naidu et al., 1991b). Together, the three studies demonstrated that lactoferrin binding is a conserved, yet strain-variable trait of *S. aureus*, mediated by specific bacterial surface proteins. About a decade later, Aguila et al. (2001), published a study on the antibacterial activity of Lf against clinical strains of *S. aureus*, in which they observed that only 13% of isolates from skin or soft-tissue infections were resistant to Lf. In contrast, half of the blood isolates showed no susceptibility to Lf, suggesting, as in studies by Naidu et al. that the bacterial-Lf interaction is strain-specific. They have also stated that the antibacterial effect of Lf was dependent on its ferrochelating properties (Aguila et al., 2001). Further molecular mechanisms of *S. aureus*-Lf interactions were described in 2008 by two separate research groups. In the study by Clarke and Foster (2008), the authors found that the *S. aureus* surface protein IsdA (a protein that is crucial for nasal colonization) binds strongly to apolactoferrin (a form of lactoferrin deprived of the iron) and protects the bacteria from its bactericidal effect. They showed that IsdA acts as a competitive inhibitor of lactoferrin’s serine protease activity, thereby reducing the killing efficiency of Lf. This work reports the existence of an immune-evasion mechanism in which *S. aureus* uses IsdA to neutralize the proteolytic activity of Lf and to survive innate host defenses (Clarke and Foster, 2008). Koprivnjak et al. (2008), further showed that specific staphylococcal cell wall alterations (e.g., wall teichoic acid deficiency) influence susceptibility to host defensins and Lf-related peptides, highlighting their relevance in innate immune interactions. Hussan et al. (2022), used human lactoferrin as an adjuvant to increase the efficacy of cefazolin against β-lactamase-producing *S. aureus* biofilms. They observed that while the MIC50 and MIC90 for cefazolin alone were 1.0 and 2.0 μg/ml, respectively, the addition of 6.25 μM lactoferrin reduced them to 0.5 μg/ml. They demonstrated that, *in vitro*, optimal concentrations of lactoferrin enhanced the activity of β-lactam antibiotics against *S. aureus* by reducing β-lactamase production (Hussan et al., 2022). These findings suggest that lactoferrin has potential as an antibiotic adjuvant against *S. aureus* in preclinical models. Together, these studies demonstrate that lactoferrin interacts with *S. aureus* through multiple, partially opposing mechanisms, including iron sequestration, direct antimicrobial effects, and bacterial evasion strategies. Antimicrobial activity of lactoferrin is therefore highly strain- and context-dependent, influenced by iron availability, bacterial surface properties, and host environmental conditions. These findings suggest that lactoferrin functions less as a universally bactericidal agent and more as a modulatory component of innate host defense whose efficacy varies across infection settings.

### 2.2 Lactoferrin-derived peptides

In addition to studies examining intact lactoferrin, several investigations have focused on lactoferrin-derived peptides, which are often more stable than the parent protein, possess distinct mechanisms of action, and may exhibit improved penetration into bacterial cells. These peptides have primarily been evaluated in *in vitro* and preclinical models, with variable activity against *S. aureus*. Xi et al. (2013), designed and characterized a hybrid peptide, LHP7, and tested its activity against clinical MRSA strains. They have observed that the peptide can actively disrupt bacterial membranes, resulting in bacterial cell death. Furthermore, a synergistic effect was observed for LHP7 with gentamycin, rifampin, tetracycline, and vancomycin (Xi et al., 2013). HLR1r peptide, a lactoferrin derivative, has Ser-Arg-Arg-Arg-Arg-Gly added to the C-terminal end to facilitate its binding to the bacterial cell wall. It was shown that the peptide exhibited antimicrobial activity against the tested MRSA strains and anti-inflammatory activity in THP-1 and MeT-5A cell lines. Furthermore, the suitability of the peptide in the treatment of skin infections was assessed using an *ex vivo* pig skin model, as well as an *in vivo* excision wound model in rats. It was observed that all tested concentrations significantly reduced the bacterial titer on the surface of the pig skin in comparison to the placebo. 2 mg/ml of HLR1r administered to MRSA-infected wounds significantly reduced the bacterial titer when compared to mupirocin (Björn et al., 2016). A year later, Hao et al. (2017), described the activity of two lactoferricin peptides, Lfcin4 and Lfcin5, against different pathogens, including *S. aureus*. They observed that treatment with Lfcin4 increased the cell surface hydrophobicity of *S. aureus* ATCC 25923 from 5.27% to 18.87%. However, treatment with Lfcin5 resulted in a non-statistically significant change in hydrophobicity, from 5.27% to 8.95%. Furthermore, treatment with 1 × MIC 0.04% for 2 h resulted in a change in cell structure (as observed under SEM) - the *S. aureus* cells appeared smaller, suggesting either growth inhibition or release of intracellular components. Viability tests conducted with proprium iodine (PI) showed an increase in damaged cells to 50.10% (compared to 0.04% in the control variant). The murine tight infection model showed that both peptides were able to significantly reduce the number of bacterial cells, though the peptide Lfcin5 was more effective (Hao et al., 2017). The two most recent studies focused on the activity of Lf-derived peptides against *S. aureus* biofilms relevant to skin and soft tissue infections. Quintieri et al. (2020), evaluated the antibiofilm activity of a bovine lactoferrin hydrolysate (HLF) and its purified peptides (lactoferricin B and lactoferrampin) against skin-borne staphylococci. They found that while the MIC of the whole HLF was relatively high (10– >20 mg/mL), its minimal biofilm inhibitory concentration was much lower (≈ 2.5 mg/mL) for most of the tested strains. Purified peptides exhibited even stronger antibiofilm activity. At the same time, a mixture of lactoferricin B and lactoferrampin had a MIC of only 0.037 mg/mL and reduced biofilm formation by >60% after 24 h of treatment (Quintieri et al., 2020). A study from 2023, by Ostrówka et al. (2023), used an *in silico* AMP-prediction tool (AmpGram) to identify three new human lactoferrin fragments (hLF 397–412, 448–464, and 668–683) with high antimicrobial activity potential. They tested the three peptides alongside the previously obtained Lf peptide hLF 1-11. Unfortunately, they found that none of the fragments exhibited strong antimicrobial activity against *S. aureus*, even when proteases were added as adjuvants (Ostrówka et al., 2023). These negative results highlight a significant challenge in using computational tools to predict antimicrobial activity: not all predicted peptides are active *in vitro*, underscoring the need for experimental validation.

Collectively, these studies indicate that lactoferrin-derived peptides can exhibit antimicrobial and antibiofilm activity against *S. aureus* in preclinical models, with membrane disruption emerging as a recurrent mechanism. However, peptide efficacy varies substantially depending on sequence, formulation, and experimental context, and *in silico* predictions do not consistently translate into biological activity. These findings suggest that lactoferrin-derived peptides are promising yet highly heterogeneous candidates, whose therapeutic potential will depend on rigorous experimental validation and optimization rather than on computational prediction alone.

### 2.3 Lactoferrin use in hydrogels

Most recently published works have provided an innovative approach: the incorporation of Lf or Lf-derived peptides into biomaterials, hydrogels, and nanoparticles to improve the antibacterial performance and regenerative properties of Lf in wound models. Zhou et al. (2024), developed a lactoferrin-eugenol hydrogel that demonstrated accelerated healing of MRSA-infected wounds in mice. They have observed that the hydrogel significantly reduced the number of bacterial colonies present at the wound site. It also promoted re-epithelialization and enhanced neovascularization by increasing HIF-1α expression at wound sites, thereby accelerating the healing process (Zhou et al., 2024). In the study by Zhang et al. (2024), the authors developed an injectable hydrogel comprising Lithium Magnesium Silicate Hydrogel (LMSH), Lactoferrin (Lf), and the antimicrobial peptide NZ2114. *In vitro* studies showed that a 1% Lf/NZ2114/LMSH gel exhibited strong antibacterial activity against *S. aureus* (≥99.99% inhibition) while maintaining ∼95% survival rate of human epidermal cells. In a murine wound infection model, the treatment with hydrogel achieved a ∼99.92% bactericidal rate and nearly 100% wound-healing rate after 14 days. The use of hydrogel also resulted in reduced IL-6 levels, restored tissue structure, promoted angiogenesis, and regenerated hair follicles and skin glands (Zhang et al., 2024). These preclinical studies, incorporating lactoferrin or lactoferrin-derived peptides into hydrogels and composite biomaterials, consistently report enhanced antibacterial activity and improved wound-healing outcomes in animal models. These delivery systems appear to mitigate some limitations of soluble lactoferrin by improving local bioavailability and enabling synergistic interactions with other antimicrobial agents. However, the diversity of materials, formulations, and experimental designs limits direct comparisons across studies, and the translational relevance of these approaches remains to be established through standardized and clinically oriented investigations.

**FIGURE 1:**
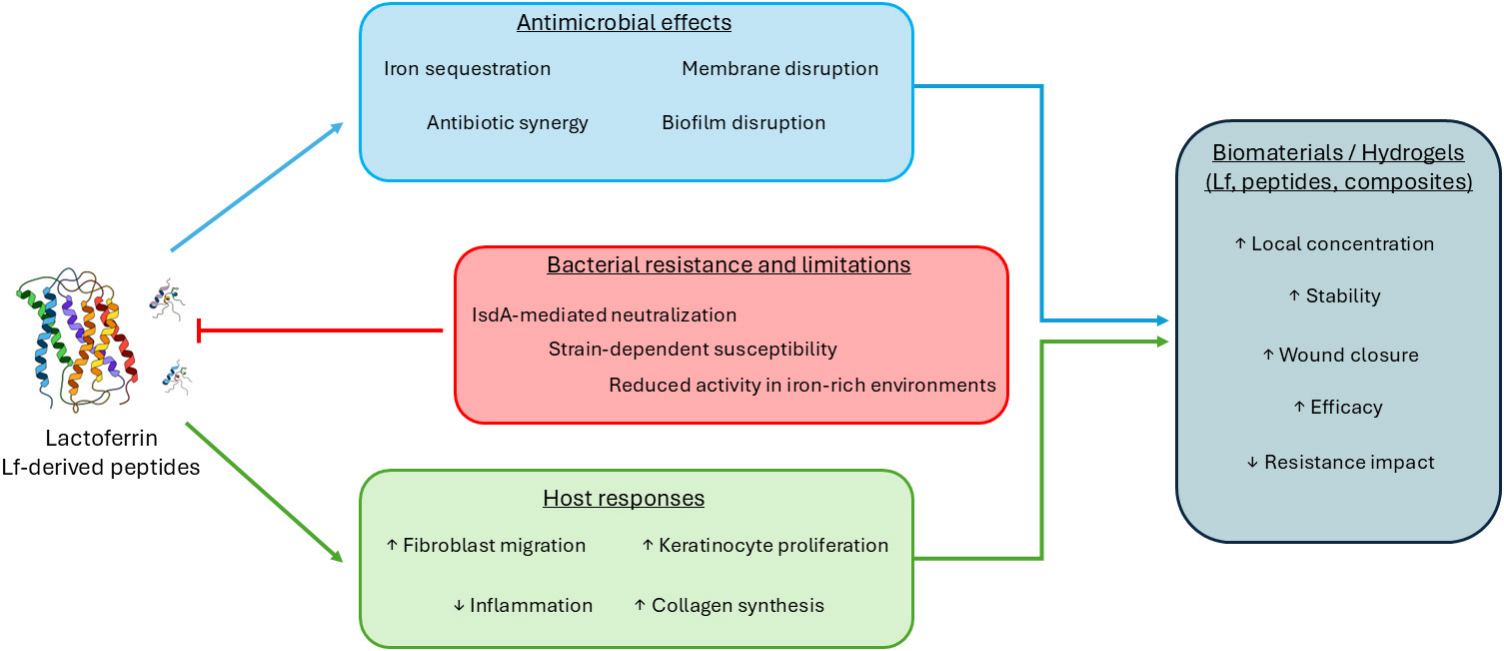
Multifactorial roles of lactoferrin and lactoferrin-derived peptides in *Staphylococcus aureus* skin and wound infections.

Together, these findings support a model in which lactoferrin exerts context-dependent antimicrobial effects that are enhanced by localized delivery systems while simultaneously promoting host wound repair processes (Figure 1).

## 3 Lactoferrin’s role in wound healing

As lactoferrin is mainly present in the human body at the mucosal membranes, i.e., in the nasopharynx, oral cavity, and conjunctival sac, it is considered primarily as a potential therapeutic to be used in the treatment of respiratory tract or eye infections (Ali et al., 2021; Regueiro et al., 2023; Kaczyńska et al., 2023). The application in the prevention of skin and wound infections is less obvious. However, lactoferrin plays a role in wound healing and skin repair processes (Takayama and Aoki, 2012). Wound healing is a complex biological process and involves multiple cell types, including immune cells, keratinocytes, fibroblasts, and endothelial cells (Tottoli et al., 2020). It has been observed that lactoferrin is involved in every stage of wound healing. Firstly, it modulates the inflammatory response of the organism in the primary stages of wound healing. It helps boost inflammation by stimulating the production of pro-inflammatory cytokines and facilitating the infiltration of immune cells into the wounded area. However, at the same time, Lf acts as an anti-inflammatory agent, preventing overreaction and allowing for further reparatory processes to begin (Engelmayer et al., 2008). Lf is also thought to facilitate granulation tissue formation by increasing the proliferation and migration of fibroblasts, which are the primary cells responsible for the generation of granulation tissue in the wound-healing process (Tang et al., 2010a; Takayama et al., 2002; Saito et al., 2011). It has also been shown that lactoferrin enhances the synthesis of extracellular matrix components, such as collagen and hyaluronan (Takayama et al., 2002; Saito et al., 2011). Furthermore, in the final stages of wound healing - re-epithelialization and wound remodeling Lf may also play a major function in the regulation of aforementioned processes. Tang et al. (2010b) have observed that the addition of lactoferrin to the medium stimulated keratinocyte proliferation and migration. It also increased the viability of starved keratinocyte cells (Tang et al., 2010b). Belvedere et al. (2021) have also observed that treatment of cells with mesoglycan/lactoferrin triggered the differentiation process of keratinocytes. Thus, beyond its well-established antimicrobial function, lactoferrin emerges as an essential contributor to the coordinated cellular events that drive effective wound healing.

## 4 Summary

Lactoferrin has emerged as a biologically versatile component of the host defense system with potential relevance for the management of *Staphylococcus aureus*–associated skin and wound infections. Over more than three decades of research, preclinical studies have demonstrated that lactoferrin and selected lactoferrin-derived peptides can inhibit bacterial growth, interfere with biofilm formation, and modulate host processes involved in tissue repair. These activities arise from a combination of antimicrobial mechanisms, immunomodulatory effects, and support of cellular functions critical for wound healing.

Recent advances in biomaterial-based delivery strategies, including hydrogels and composite dressings, have further highlighted the potential of lactoferrin-based approaches by improving local bioavailability and combining antibacterial effects with enhanced wound regeneration in animal models. Collectively, the studies reviewed here support the use of lactoferrin as an adjuvant to improve the efficacy of existing antimicrobial therapies, particularly in the context of rising antibiotic resistance.

At the same time, the available evidence also emphasizes important limitations. Lactoferrin activity against *S. aureus* is strain- and context-dependent, with bacterial resistance mechanisms attenuating its effects. Furthermore, most data derive from *in vitro* or animal studies. Consequently, the clinical utility of lactoferrin-based interventions for infected wounds remains to be established. Future progress will depend on standardized experimental designs, optimized formulations, and well-controlled translational studies to clarify the realistic therapeutic role of lactoferrin in modern wound care.
